# Correction: Microtubule reorganization during female meiosis in *C. elegans*

**DOI:** 10.7554/eLife.86893

**Published:** 2023-02-23

**Authors:** Ina Lantzsch, Che-Hang Yu, Yu-Zen Chen, Vitaly Zimyanin, Hossein Yazdkhasti, Norbert Lindow, Erik Szentgyoergyi, Ariel M Pani, Steffen Prohaska, Martin Srayko, Sebastian Fürthauer, Stefanie Redemann

**Keywords:** *C. elegans*

Lantzsch I, Yu C-H, Chen Y-Z, Zimyanin V, Yazdkhasti H, Lindow N, Szentgyoergyi E, Pani AM, Prohaska S, Srayko M, Fürthauer S, Redemann S. 2021. Microtubule reorganization during female meiosis in *C. elegans*. *eLife* **10**:e58903. doi: 10.7554/eLife.58903.

Published 11 June 2021

We discovered that the shown Length distribution of microtubules composing the different spindles for Meiosis II shown in Figure 1D was scaled wrongly. This must have happened during the combination of the Meiosis I and Meiosis II dataset into a single graph. We corrected the scaling, which does not change the overall shape of the length distribution as well as any of the quantifications and results, but does affect the visual representation of data presented in Figure 1D.

We also noticed that Figure 8 and Figure 8—figure supplement 1 showed the same image. The wrong Figure 8 was included in the final published version of the article owing to a production error.

The corrected Figure 1 is shown here:

**Figure 1:**
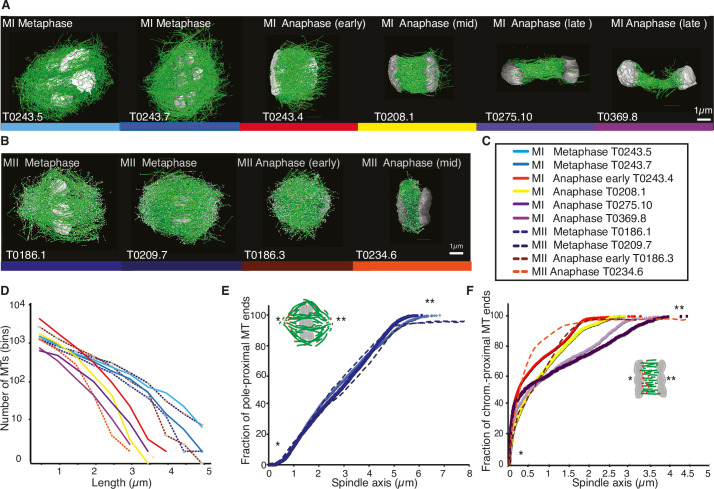


The originally published Figure 1 is shown for reference:

**Figure 2:**
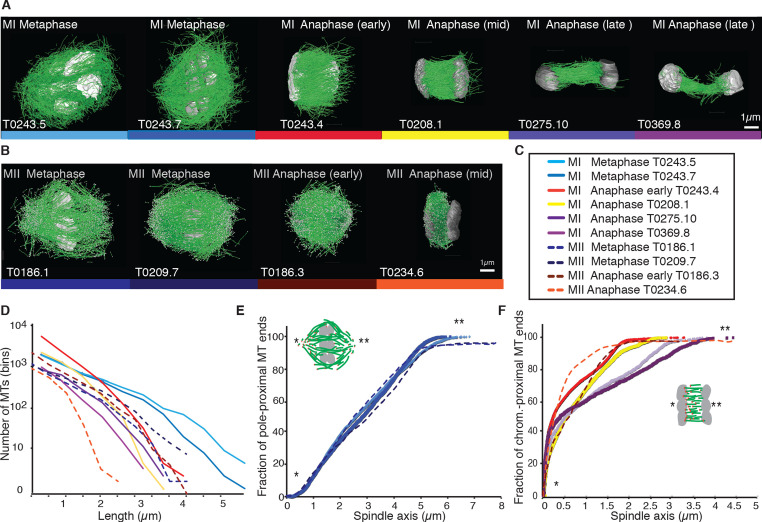


The corrected Figure 8 is shown here:

**Figure 3:**
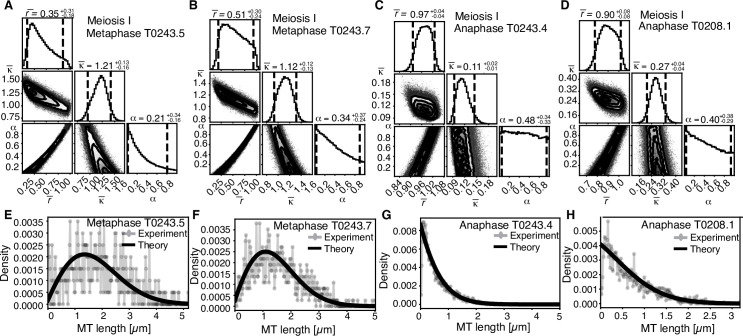


The originally published Figure 8 is shown here for comparison:

**Figure 4:**
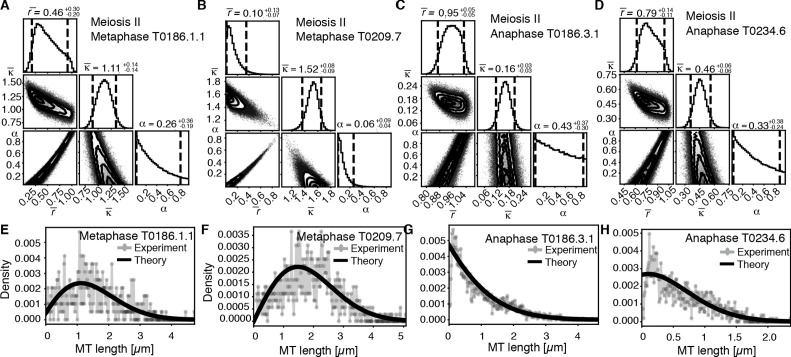


The article has been corrected accordingly.

